# Evaluation of the Hemoglobin, Albumin, Lymphocyte, and Platelet Score and Inflammatory Markers in Preterm Premature Rupture of Membranes

**DOI:** 10.1111/aji.70303

**Published:** 2026-07-25

**Authors:** Çiğdem Akçabay, Dilara Duygulu Bulan, Batuhan Tepe, Melisa Golgelioglu, Salih Burçin Kavak

**Affiliations:** ^1^ Department of Obstetrics and Gynecology, Faculty of Medicine Fırat University Elazığ Türkiye; ^2^ Department of Perinatology Ankara Etlik City Hospital Ankara Türkiye; ^3^ Department of Obstetrics and Gynecology Elazığ Fethi Sekin City Hospital Elazığ Türkiye; ^4^ Department of Obstetrics and Gynecology Yozgat City Hospital Yozgat Türkiye

**Keywords:** gestational age, hemoglobin albumin lymphocyte platelet score, inflammatory markers, neonatal outcome, preterm premature rupture of membranes

## Abstract

**Problem:**

To evaluate the hemoglobin, albumin, lymphocyte, and platelet (HALP) score and its relationship with inflammatory markers in patients with preterm premature rupture of membranes (PPROM), and to assess their association with gestational age and neonatal outcomes.

**Method of Study:**

This retrospective study included 226 pregnant women diagnosed with PPROM between 24 and 34 weeks of gestation between 2023 and 2026. Patients were categorized according to gestational age at diagnosis as early PPROM (24 + 0 to < 32 + 0 weeks) and late PPROM (32 + 0 to 34 + 0 weeks). Demographic, clinical, and laboratory data were collected, including C‐reactive protein (CRP), neutrophil‐to‐lymphocyte ratio (NLR), platelet‐to‐lymphocyte ratio (PLR), and systemic immune‐inflammation index (SII). The HALP score was calculated using routine laboratory parameters. Receiver operating characteristic (ROC) analysis was performed to evaluate the ability of the HALP score and inflammatory markers to identify early PPROM. Multivariate logistic regression analysis was used to identify independent predictors of adverse neonatal outcomes.

**Results:**

Patients in the early PPROM group delivered at significantly earlier gestational ages, had lower birth weights, and had longer latency periods than those in the late PPROM group (all *p* < 0.001). Inflammatory markers, including CRP, NLR, PLR, and SII, were higher in the early PPROM group, whereas hemoglobin, lymphocyte levels, and HALP scores were lower (all *p* < 0.05). Adverse neonatal outcomes were more frequent in the early PPROM group than in the late PPROM group (53.5% vs. 20.6%, *p* < 0.001). ROC analysis demonstrated that CRP had the highest diagnostic performance (AUC: 0.663), followed by SII and NLR, whereas HALP showed modest discriminative ability (AUC: 0.632). In multivariate analysis, only gestational age at diagnosis was identified as an independent predictor of adverse neonatal outcome.

**Conclusion:**

The HALP score and inflammatory markers were associated with earlier gestational age at PPROM diagnosis, suggesting a relationship with inflammatory and immunonutritional status. However, their ability to predict adverse neonatal outcomes was limited, and gestational age remained the primary determinant of neonatal prognosis. Therefore, HALP may be considered a complementary biomarker rather than a standalone prognostic tool.

**Abbreviations**
CRP: C‐reactive protein
CI: confidence interval
FGR: fetal growth restriction
g: grams
g/dL: grams per deciliter
GW: gestational week
HALP: hemoglobi, albumin, lymphocyte, platelet
OR: odds ratio
SE: standard error
PPROM: preterm premature rupture of membranes
PLR: platelet‐to‐lymphocyte ratio
SII: systemic immune‐inflammation index
mg/L: milligrams per liter
min: minute.
NLR: neutrophil‐to‐lymphocyte ratio
NP index: neutrophil‐to‐platelet index
×10^3^/µL: ×10^3^ per microliter

## 1 Introduction

Preterm premature rupture of membranes (PPROM) is a significant obstetric complication that is associated with increased maternal, fetal, and neonatal morbidity and mortality [1]. It is linked to a number of pathophysiological mechanisms, most notably intrauterine infection and inflammatory processes [2]. Early identification of patients at high risk for adverse outcomes remains a significant clinical challenge. Pro‐inflammatory cytokines and matrix metalloproteinase enzyme activation contribute to the disruption of fetal membrane integrity and lead to the onset of disease [3]. In recent years, systemic inflammatory markers derived from standard laboratory measures have garnered interest as potential predictors of obstetric problems [4]. While the pivotal function of inflammation in the onset of PPROM is clearly established, the identification of reliable biomarkers to predict clinical course and possible complications of the condition continues to be a significant clinical necessity. C‐reactive protein (CRP) is a commonly utilized biomarker for inflammation and infection; nevertheless, its specificity in prediction of PPROM‐related outcomes is limited [5]. Similarly, hematological parameters such as neutrophil‐lymphocyte ratio (NLR), platelet‐lymphocyte ratio (PLR), and systemic immune‐inflammatory index (SII) stand out as inexpensive and easily accessible markers reflecting the balance between inflammation and immune response [6]. These indices have demonstrated diagnostic and prognostic significance in numerous obstetric diseases, especially in conditions characterized by significant inflammation [7]. Nevertheless, relying just on these indicators might fail to sufficiently represent the complex nature of inflammation.

Recently, composite scores, which combine both hematological and biochemical parameters, have been proposed as indicators of systemic inflammation and nutritional status [8]. The HALP score has been increasingly evaluated as an immunonutritional biomarker because it integrates hemoglobin, albumin, lymphocyte, and platelet levels, thereby reflecting anemia, nutritional reserve, immune status, and platelet‐related inflammatory activity within a single index [9]. Although most early evidence regarding HALP originated from oncology and other systemic inflammatory conditions [10, 11, 12], recent obstetric studies have suggested that HALP may also be associated with placental dysfunction‐related disorders and adverse perinatal outcomes. In pregnancies complicated by PPROM, recent evidence has further indicated that the SII may have greater predictive value than HALP for neonatal outcomes and delivery timing, supporting the need for additional studies evaluating these markers in different PPROM cohorts [7, 13].

Considering that PPROM is marked by inflammatory activation, modified immune responses, and compromised tissue integrity, assessing the HALP score alongside well‐defined inflammatory markers like CRP, NLR, SII, and PLR can yield a more thorough understanding of the disease's biological behavior and clinical progress. This combined assessment may provide additional insight into the biological background of PPROM, although its direct clinical utility requires further validation.

This study aimed to evaluate the HALP score in patients with PPROM and to investigate its association with inflammatory markers, gestational age at diagnosis, neonatal outcomes, and clinical course.

## 2 Materials and Methods

### 2.1 Study Design and Population

This retrospective study was conducted in the Department of Obstetrics and Gynecology at Fırat University between January 2023 and January 2026. Medical records of patients diagnosed with preterm premature rupture of membranes (PPROM) were reviewed using hospital electronic databases and patient files. The study protocol was approved by the Non‐Interventional Research Ethics Committee of Fırat University (Approval No: 2026/07‐19). Due to the retrospective design of the study, informed consent was waived.

PPROM was defined as rupture of membranes before 37 weeks of gestation prior to the onset of labor. However, only patients diagnosed with PPROM between 24 + 0 and 34 + 0 weeks of gestation were included in the present study. The diagnosis was based on the clinical observation of active amniotic fluid leakage during sterile speculum examination. Chorioamnionitis was evaluated based only on clinical findings.

A total of 226 pregnant women diagnosed with PPROM between 24 + 0 and 34 + 0 weeks of gestation were included in the study. Only patients diagnosed with PPROM between 24 + 0 and 34 + 0 weeks of gestation were included. Patients beyond 34 weeks were excluded because current clinical management generally favors delivery rather than expectant management after this gestational age. Consequently, these pregnancies have little or no clinically meaningful latency period, limiting the opportunity for inflammation‐related maternal and neonatal complications to develop. Restricting the study population to pregnancies managed expectantly before 34 weeks therefore provided a more appropriate cohort for evaluating the association of the HALP score with inflammatory status, latency period, and adverse neonatal outcomes.

Patients were categorized into two groups according to gestational age at PPROM diagnosis: early PPROM (24 + 0 to <32 + 0 weeks) and late PPROM (32 + 0 to 34 + 0 weeks).

This 32‐week threshold was selected as a clinically relevant cut‐off in PPROM management, particularly in relation to fetal maturation and neuroprotective considerations [14].

Patients with multiple pregnancies, known fetal anomalies, intrauterine fetal demise, or incomplete medical records were excluded from the study. In addition, patients with maternal systemic diseases, including chronic inflammatory conditions, autoimmune disorders, or active infections that could affect systemic inflammatory parameters, were not included in the analysis. Patients diagnosed with PPROM before 24 + 0 weeks or after 34 + 0 weeks of gestation were also excluded.

### 2.2 Data Collection

Demographic, obstetric, and clinical data were retrospectively obtained from hospital records. All laboratory parameters were obtained at the time of hospital admission, before the administration of corticosteroids, antibiotics, or other medical treatments that could affect systemic inflammatory markers.

Laboratory parameters, including hemoglobin, platelet count, neutrophil count, lymphocyte count, albumin, and CRP, were recorded. In addition, derived inflammatory indices such as the neutrophil‐to‐lymphocyte ratio (NLR), platelet‐to‐lymphocyte ratio (PLR), and systemic immune‐inflammation index (SII) were calculated. The SII was calculated as platelet count × neutrophil count / lymphocyte count. The HALP score was calculated as hemoglobin (g/L) × albumin (g/L) × lymphocyte count (10^9^/L) / platelet count (10^9^/L).

Adverse neonatal outcome was defined as the presence of at least one of the following conditions: A 5 min Apgar score of less than 7 or an umbilical cord pH value below 7.20.

### 2.3 Statistical Analyses

Statistical analyses were performed using SPSS software (version 22, IBM Corp., Armonk, NY, USA). The distribution of continuous variables was assessed using the Kolmogorov–Smirnov test. Since the variables were not normally distributed, continuous variables were expressed as median (Q1‐Q3) and compared using the Mann–Whitney *U* test. Categorical variables were presented as number (percentage) and compared using the Chi‐square test or Fisher's exact test, as appropriate.

Receiver operating characteristic (ROC) curve analysis was performed to evaluate the ability of HALP score, CRP, NLR, PLR, and SII to identify early PPROM (< 32 weeks). The area under the curve (AUC) was calculated, and optimal cut‐off values were determined using the Youden index. For HALP score, which was lower in the early PPROM group, ROC analysis was interpreted after reversing the direction of the test variable, so that lower HALP values indicated early PPROM.

Univariate logistic regression analysis was initially performed to identify variables associated with adverse neonatal outcome. Variables with a *p*‐value < 0.10 in univariate analysis were subsequently included in the multivariate logistic regression model to determine independent predictors. Results were expressed as odds ratios (ORs) with 95% confidence intervals (CIs). Additionally, Spearman's rank correlation analysis was performed to evaluate the relationship between gestational age at PPROM diagnosis and clinical, obstetrical, and laboratory parameters across the overall cohort. A two‐tailed *p*‐value < 0.05 was considered statistically significant.

## 3 Results

A total of 226 patients diagnosed with PPROM between 24 + 0 and 34 + 0 weeks of gestation were included in the study, of whom 129 (57.1%) were in the early PPROM group and 97 (42.9%) were in the late PPROM group.

There was no significant difference between the groups in terms of maternal age (*p* = 0.446). However, patients in the early PPROM group delivered at significantly earlier gestational ages and had lower birth weights than those in the late PPROM group, whereas the latency period was significantly longer in the early PPROM group (all *p* < 0.001). Among obstetric complications, fetal growth restriction and oligohydramnios were more frequent in the early PPROM group (*p* = 0.009 and *p* < 0.001, respectively), while other maternal comorbidities were similar between the groups (Table 1).

**TABLE 1:** Maternal and obstetric characteristics based on gestational age at the diagnosis of PPROM.

Variable	Early PPROM (*n* = 129)	Late PPROM (*n* = 97)	*p*
Maternal age (years)	29 (25–33)	29 (23–34)	0.446^a^
GA at diagnosis (weeks)	28 (26–30)	33 (32–33)	< 0.001^a^
Birth weight (g)	1235 (832–1800)	2100 (1820–2300)	< 0.001^a^
Latency period (days)	4 (0–16.5)	1 (0–4)	< 0.001^a^
GA at delivery (weeks)	29.7 (27.3–31.5)	33.5 (33.1–34.0)	< 0.001^a^
Hypertension	2 (1.6)	3 (3.1)	0.654^b^
Gestational diabetes	6 (4.7)	8 (8.2)	0.280^b^
FGR	4 (3.1)	12 (12.4)	0.009^b^
Urinary tract infection	5 (3.9)	2 (2.1)	0.702^b^
Oligohydramnios	57 (44.2)	17 (17.5)	< 0.001^c^
Cervical dilation	65 (50.4)	50 (51.4)	0.863^c^

*Note:* Values are presented as median (Q1‐Q3) for continuous variables and *n* (%) for categorical variables.

Early PPROM was defined as PPROM diagnosed between 24 + 0 and < 32 + 0 weeks of gestation, whereas late PPROM was defined as PPROM diagnosed between 32 + 0 and 34 + 0 weeks of gestation.

Abbreviations: GA, gestational age; FGR, fetal growth restriction.

^a^ Mann–Whitney *U* test.

^b^ Fisher's exact test.

^c^ Pearson chi‐square test.

Laboratory analysis demonstrated that inflammatory markers were significantly elevated in the early PPROM group. Neutrophil count, CRP, NLR, PLR, and SII levels were all significantly higher, whereas lymphocyte count and hemoglobin levels were significantly lower in early PPROM (all *p* < 0.05). The HALP score was significantly lower in the early PPROM group compared to the late PPROM group (*p* = 0.001) (Table 2).

**TABLE 2:** Laboratory parameters and inflammatory indices according to gestational age at PPROM diagnosis.

Variable	Early PPROM (*n* = 129)	Late PPROM (*n* = 97)	*p*
Hemoglobin (g/dL)	11.50 (10.50–12.43)	12.00 (10.80–12.83)	0.027^a^
Platelet (× 10^3^/µL)	255.00 (219.75–297.00)	253.00 (195.75–286.25)	0.425^a^
Neutrophil (× 10^3^/µL)	10.30 (7.57–13.13)	8.40 (6.93–10.98)	0.001^a^
Lymphocyte (× 10^3^/µL)	1.60 (1.24–2.18)	1.90 (1.45–2.46)	0.007^a^
Albumin (g/dL)	3.80 (3.60–4.10)	3.90 (3.70–4.00)	0.701^a^
CRP (mg/L)	9.90 (4.12–19.24)	5.20 (3.30–9.75)	< 0.001^a^
NLR	5.80 (4.20–9.53)	4.50 (3.24–6.43)	< 0.001^a^
PLR	148.20 (115.74–210.82)	123.50 (98.81–174.04)	0.003^a^
SII	1498.50 (987.07–2345.27)	1007.60 (736.26–1634.47)	< 0.001^a^
NP index	38.00 (25.00–48.00)	39.00 (25.00–45.00)	0.499^a^
HALP score	29.53 (20.87–39.04)	36.05 (25.77–46.66)	0.001^a^

*Note:* Values are presented as median (Q1‐Q3).

Group definitions are the same as those described in Table 1.

Abbreviations: CRP, C‐reactive protein; NLR, neutrophil‐to‐lymphocyte ratio; PLR, platelet‐to‐lymphocyte ratio; SII, systemic immune‐inflammation index; NP index, neutrophil‐to‐platelet index; HALP, hemoglobin, albumin, lymphocyte, and platelet score.

^a^ Mann–Whitney *U* test.

Regarding maternal and neonatal outcomes, placental abruption and cord prolapse were significantly more frequent in the early PPROM group (*p* = 0.013 and *p* = 0.003, respectively). Neonates in this group also had significantly lower 1‐ and 5‐min Apgar scores (both *p* < 0.001), whereas umbilical cord pH values did not differ significantly between the groups (*p* = 0.766). Accordingly, adverse neonatal outcomes were significantly more common in the early PPROM group (53.5% vs. 20.6%, *p* < 0.001) (Table 3).

**TABLE 3:** Maternal and neonatal outcomes according to gestational age at PPROM diagnosis.

Variable	Early PPROM (*n* = 129)	Late PPROM (*n* = 97)	*p*
Chorioamnionitis	7 (5.4)	1 (1.0)	0.142^a^
Placental abruption	18 (14.0)	4 (4.1)	0.013^a^
Cord prolapse	11 (8.5)	0 (0.0)	0.003^a^
Apgar score (1 min)	5.00 (0.00–9.00)	6.00 (2.00–9.00)	< 0.001^b^
Apgar score (5 min)	7.00 (0.00–10.00)	8.00 (5.00–10.00)	< 0.001^b^
Umbilical cord pH	7.33 (6.30–7.39)	7.31 (6.80–7.38)	0.766^b^
Adverse neonatal outcome	69 (53.5)	20 (20.6)	< 0.001^a^

*Note:* Values are presented as median (Q1‐Q3) for continuous variables and *n* (%) for categorical variables.

PPROM, preterm premature rupture of membranes.

Group definitions are the same as those described in Table 1.

^a^ Fisher's exact test.

^b^ Mann–Whitney *U* test.

When patients were stratified according to adverse neonatal outcome, those with adverse outcomes had significantly lower gestational age at diagnosis and birth weight (both *p* < 0.001). However, latency period, CRP levels, HALP score, and oligohydramnios were not significantly different between groups (all *p* > 0.05) (Table 4).

**TABLE 4:** Comparison according to adverse neonatal outcome.

Variable	No adverse outcome (*n* = 137)	Adverse outcome (*n* = 89)	*p*
Gestational age at diagnosis (weeks)	33.00 (31.0–34.0)	29.20 (25.60–32.40)	< 0.001^a^
Birth weight (g)	1900.00 (1396.00–2235.00)	1127.00 (750.00–1802.00)	< 0.001^a^
Latency period (days)	2.00 (0.00–9.00)	3.00 (0.00–9.00)	0.188^a^
Oligohydramnios	39 (28.5)	35 (39.3)	0.089^b^
CRP (mg/L)	6.10 (3.30–12.30)	9.80 (3.60–19.90)	0.151^a^
HALP score	33.50 (22.60–43.20)	30.10 (22.70–43.10)	0.135^a^

*Note:* Values are presented as median (Q1‐Q3) for continuous variables and n (%) for categorical variables.

Abbreviations: CRP, C‐reactive protein; HALP, hemoglobin, albumin, lymphocyte, and platelet score.

^a^ Mann–Whitney *U* test.

^b^ Chi‐square test.

**FIGURE 1:**
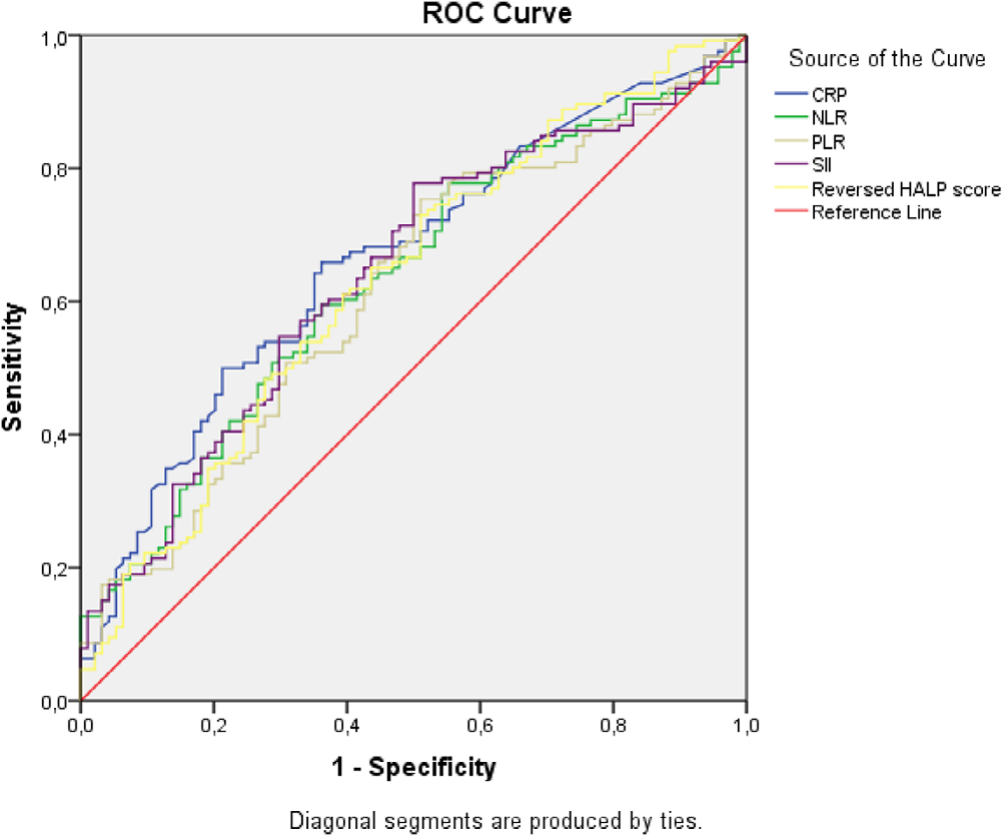
Receiver operating characteristic curves of inflammatory markers and reversed HALP score for identifying early PPROM. Early PPROM was defined as PPROM diagnosed between 24 +0 and < 32 + 0 weeks of gestation.

ROC curve analysis demonstrated modest discriminatory performance for identifying early PPROM. CRP showed the highest AUC value (AUC: 0.663, 95% CI: 0.591–0.734, *p* < 0.001), with an optimal cut‐off of ≥ 6.35 mg/L, sensitivity of 65.9%, and specificity of 63.8%. The optimal cut‐off values for NLR, PLR, and SII were ≥ 5.08, ≥ 116.30, and ≥ 977.60, yielding sensitivities of 59.5%, 75.4%, and 77.8%, and specificities of 63.8%, 48.9%, and 50.0%, respectively. After reversing the direction of HALP because lower values were associated with early PPROM, HALP demonstrated modest discriminatory ability (AUC: 0.632, 95% CI: 0.558–0.706, *p* = 0.001), with an optimal cut‐off of ≤ 38.26, sensitivity of 73.0%, and specificity of 48.9% (Figure 1).

Spearman correlation analysis performed across the overall cohort revealed that gestational age at PPROM diagnosis was significantly negatively correlated with CRP (*ρ* = −0.28, *p* < 0.001), NLR (*ρ* = −0.25, *p* < 0.001), SII (*ρ* = −0.23, *p* < 0.001), and PLR (*ρ* = −0.18, *p* = 0.007). A significant positive correlation was observed between gestational age and HALP score (*ρ* = + 0.22, *p* = 0.001) and lymphocyte count (*ρ* = + 0.17, *p* = 0.010). Albumin and platelet count did not demonstrate a statistically significant correlation with gestational age at PPROM diagnosis (*p* > 0.05 for both) (Table 5).

**TABLE 5:** Spearman correlation analysis between gestational age at PPROM diagnosis and clinical parameters.

Variable	Spearman *ρ*	*p* value
CRP (mg/L)	−0.28	< 0.001
NLR	−0.25	< 0.001
SII	−0.23	< 0.001
PLR	−0.18	0.007
HALP score	+0.22	0.001
Lymphocyte (× 10^3^/µL)	+0.17	0.010
Hemoglobin (g/dL)	+0.14	0.034
Albumin (g/dL)	+0.03	0.655
Platelet (× 10^3^/µL)	+0.05	0.445

Abbreviations: CRP, C‐reactive protein; NLR, neutrophil‐to‐lymphocyte ratio; PLR, platelet‐to‐lymphocyte ratio; SII, systemic immune‐inflammation index; HALP, hemoglobin, albumin, lymphocyte, and platelet score; PPROM, preterm premature rupture of membranes. Spearman's rank correlation coefficient (ρ) reflects the monotonic relationship between each parameter and gestational age at PPROM diagnosis across the overall cohort.

In multivariate logistic regression analysis, which included gestational age at diagnosis, HALP score, CRP levels, oligohydramnios, placental abruption, and cord prolapse, only gestational age at diagnosis was identified as an independent predictor of adverse neonatal outcome (OR: 0.77, 95% CI: 0.69–0.87, *p* < 0.001). The remaining variables were not found to be significant predictors (all *p* > 0.05) (Table 6).

**TABLE 6:** Multivariate logistic regression analysis for predicting adverse neonatal outcome.

Variable	OR (95% CI)	*p*
Gestational age at diagnosis (week)	0.77 (0.69–0.87)	< 0.001
HALP score	1.11 (0.19–6.60)	0.906
CRP (mg/L)	1.00 (0.99–1.01)	0.875
Oligohydramnios	1.27 (0.63–2.55)	0.509
Placental abruption	2.17 (0.78–6.03)	0.138
Cord prolapse	1.99 (0.50–7.99)	0.333

Abbreviations: OR, odds ratio; CI, confidence interval; HALP, hemoglobin, albumin, lymphocyte, and platelet score; CRP, C‐reactive protein.

## 4 Discussion

In this cohort of patients diagnosed with preterm premature rupture of membranes between 24 + 0 and 34 + 0 weeks of gestation, 57.1% were diagnosed before 32 weeks, whereas 42.9% were diagnosed between 32 + 0 and 34 + 0 weeks. Oligohydramnios was identified in 32.7% of cases, fetal growth restriction in 7.1%, placental abruption in 9.7%, cord prolapse in 4.9%, and chorioamnionitis in 3.5%. In the early PPROM group, gestational age at delivery and birth weight were lower, whereas the latency period was longer (4 vs. 1 day) (all *p* < 0.001). Inflammatory markers, including CRP, NLR, PLR, and SII, were markedly elevated in the early PPROM group, but hemoglobin, lymphocyte count, and HALP score were decreased (all *p* < 0.05). Adverse neonatal outcomes were significantly more frequent in the early PPROM group (53.5% vs. 20.6%, *p* < 0.001). ROC analysis revealed limited discriminatory performance for all markers, with CRP showing the highest AUC (0.663), while HALP demonstrated comparable but modest discriminatory ability (AUC: 0.632). Spearman correlation analysis revealed weak‐to‐moderate associations between gestational age at PPROM diagnosis and inflammatory markers (*ρ* ranging from −0.18 to −0.28) and HALP score (*ρ* = + 0.22), suggesting that these parameters are biologically related to gestational age but capture only a limited portion of its variability. This may explain their modest discriminatory performance in ROC analysis and inability to independently predict adverse neonatal outcomes.

This study revealed that inflammatory markers, including CRP, NLR, PLR, and SII, were considerably elevated in the early PPROM group, suggesting a heightened inflammatory response during the midpregnancy. Studies have shown that inflammation plays a central role in the pathophysiology of PPROM and is related to disease severity [4, 15]. Notably, FGR was more prevalent in the late PPROM group, suggesting that placental vascular dysfunction may play a more prominent role in late PPROM, whereas ascending infection and inflammation appear to be the predominant mechanisms in early PPROM. NLR and SII are recognized as reliable indices of inflammatory activation in PPROM, correlating with a reduced latent period and an elevated risk of complications [2, 11]. High CRP levels are similarly linked to intrauterine infection and negative perinatal outcomes; however, it is noted that their specificity is limited [16]. Our findings indicate a substantial elevation in inflammatory markers in early PPROM cases. In addition, the early PPROM group demonstrated significantly higher rates of placental abruption and cord prolapse, which may have further contributed to the adverse neonatal outcomes observed in this group.

Unlike classical inflammatory indices, the HALP score provides indirect information not only about the inflammatory response based on leukocytes, lymphocytes, or platelets, but also about the patient's oxygen‐carrying capacity, nutritional status, and overall physiological reserve through its hemoglobin and albumin components [16, 17]. In this context, HALP is regarded as a composite biomarker facilitating the simultaneous evaluation of inflammation and immune‐nutritional status. The literature reports that low HALP scores are associated with poor prognosis in various malignant and inflammatory diseases [11]. Furthermore, HALP and similar nutritional‐inflammatory indices have recently attracted interest in obstetrics as potential markers of placental dysfunction and adverse perinatal outcomes [12]. Bozkurt Ozdal et al. [7]. demonstrated that lower first‐trimester HALP scores were associated with placental abruption and adverse perinatal outcomes, including intrauterine fetal death and neonatal intensive care unit admission. These findings suggest that HALP may reflect the inflammatory, hematologic, and nutritional alterations associated with placental dysfunction‐related disorders and support its potential relevance in obstetric risk assessment.

The lower HALP score found in early PPROM cases in this study suggests that early PPROM may be associated not only with increased inflammatory activity but also with lower immune‐nutritional reserves. However, the inability of the HALP score to independently predict adverse neonatal outcomes suggests that gestational age is the dominant determinant of neonatal prognosis, and that HALP should be considered more as an auxiliary marker reflecting the biological/inflammatory basis of the disease. Recent evidence has increasingly focused on evaluating composite inflammatory indices, including HALP and SII, in pregnancies complicated by PPROM [13, 18]. Emeklioglu et al. [13]. reported that higher SII values were associated with lower birth weight, and lower 1‐ and 5 min Apgar scores, whereas HALP showed limited and inconsistent predictive performance and was not significantly associated with adverse outcomes. These findings are largely consistent with our results, in which HALP was lower in early PPROM but did not independently predict adverse neonatal outcome.Taken together, these findings suggest that HALP may reflect the inflammatory and immunonutritional background of PPROM, but its standalone prognostic value for neonatal outcomes appears limited.

The observation that CRP exhibited the highest  discriminatory performance in ROC analysis aligns with its ability to indicate acute inflammation [3]. The comparable discriminating power of the HALP score (AUC: 0.632) supports the potential of this composite indicator to represent biological processes in PPROM. The HALP score, predominantly utilized in the literature for malignant and vascular disorders, may serve as an additional marker in intricate obstetric conditions as PPROM in this study, due to its nature that encompasses both inflammatory and immune‐nutritional parameters [9, 10].

In multivariate analysis, the HALP score and other inflammatory markers were not found to be independent predictors of adverse neonatal outcomes, while only gestational age at diagnosis was found to be significant, highlighting the decisive role of prematurity in determining neonatal prognosis [2]. This indicates that whereas inflammatory mechanisms significantly contribute to the development and molecular bases of PPROM, newborn outcomes are chiefly associated with the degree of fetal maturation. Consequently, whereas inflammatory indicators indicate disease severity and biological activity, they may be inadequate alone for predicting clinical outcomes.

Clinically, a low HALP score in PPROM patients can be seen as a supportive marker indicating not only an increased inflammatory burden but also a decreased oxygen‐carrying capacity and weakened immune‐nutritional reserves [19, 20]. In pregnant women with low HALP scores, a closer assessment of the patient's nutritional status may be considered in addition to standard approaches. Therefore, the HALP score can be used as a practical tool regarding the patient's overall physiological condition, rather than being a sole decision‐maker.

The fact that adverse neonatal outcomes were defined in this study solely based on low 5 min Apgar scores and umbilical cord pH values may have contributed to the seemingly limited prognostic value of the HALP score. Given that the HALP score integrates parameters related to inflammation, oxygen‐carrying capacity, and nutritional reserve, its clinical relevance may be more evident in neonatal morbidities with a stronger inflammatory component, including neonatal sepsis, neonatal intensive care unit admission, respiratory distress syndrome, and necrotizing enterocolitis. Accordingly, further studies evaluating broader composite neonatal outcomes are warranted to more clearly determine the prognostic utility of the HALP score in PPROM.

Interestingly, latency period was significantly longer in patients diagnosed before 32 weeks. This finding may reflect the greater tendency toward expectant management in earlier gestational ages despite the presence of increased inflammatory burden. Future studies evaluating the relationship between HALP score, inflammatory markers, and short latency intervals may provide further insight into the biological mechanisms underlying PPROM progression.

The retrospective design of the study limits the establishment of causal relationships and may be susceptible to potential data deficiencies and recording errors. Furthermore, being conducted in a single center may limit the generalizability of the findings. While the sample size is moderate, it should be considered that the statistical power may be limited, especially in terms of subgroup analyses. Additionally, the limited number of FGR cases (*n* = 16) precluded a reliable assessment of the HALP score's discriminatory performance for identifying FGR. Nevertheless, given the limited data in the literature regarding the role of HALP score in PPROM, the findings of this study are instructive for future larger, multicenter, and prospective studies.

In conclusion, the HALP score and systemic inflammatory markers were associated with earlier gestational age at PPROM diagnosis, suggesting that early PPROM may be accompanied by a heightened inflammatory burden and diminished immunonutritional reserve. Nonetheless, these markers did not independently predict adverse neonatal outcomes, with gestational age remaining the primary contributor. Therefore, HALP may be considered a complementary biomarker reflecting the biological background of PPROM rather than an independent prognostic marker. Further prospective studies incorporating broader neonatal morbidity outcomes are needed to clarify its clinical utility.

## Conflicts of Interest

The authors declare that they have no conflicts of interest.

## Ethics Statement

The study protocol was approved by the Non‐Interventional Research Ethics Committee of Fırat University with the approval number 2026/07‐19. The study was conducted in accordance with the principles of the Declaration of Helsinki. Due to the retrospective design of the study, the requirement for informed consent was waived by the ethics committee.
